# Modelling the dynamic basic reproduction number of dengue based on MOI of *Aedes albopictus* derived from a multi-site field investigation in Guangzhou, a subtropical region

**DOI:** 10.1186/s13071-024-06121-y

**Published:** 2024-02-21

**Authors:** Xiang Guo, Li Li, Wenwen Ren, Minling Hu, Ziyao Li, Shu Zeng, Xiaohua Liu, Yuji Wang, Tian Xie, Qingqing Yin, Yuehong Wei, Lei Luo, Benyun Shi, Chunmei Wang, Rangke Wu, Zhicong Yang, Xiao-Guang Chen, Xiaohong Zhou

**Affiliations:** 1 Department of Pathogen Biology, Institute of Tropical Medicine, Key Laboratory of Prevention and Control for Emerging Infectious Diseases of Guangdong Higher Institutes, Guangdong Provincial Key Laboratory of Tropical Disease Research, School of Public Health, Southern Medical University, Guangzhou, 510515 China; 2 State Key Laboratory of Organ Failure Research, Department of Biostatistics, Guangdong Provincial Key Laboratory of Tropical Disease Research, School of Public Health, Southern Medical University, Guangzhou, China; 3 Guangzhou Center for Disease Control and Prevention, Guangzhou, China; 4 School of Computer Science and Technology, Nanjing Tech University, Nanjing, China; 5 The School of Foreign Studies, Southern Medical University, Guangzhou, China

**Keywords:** *Aedes albopictus*, Dengue, Surveillance system, Basic reproduction number

## Abstract

**Background:**

More than half of the global population lives in areas at risk of dengue (DENV) transmission. Developing an efficient risk prediction system can help curb dengue outbreaks, but multiple variables, including mosquito-based surveillance indicators, still constrain our understanding. Mosquito or oviposition positive index (MOI) has been utilized in field surveillance to monitor the wild population density of *Aedes albopictus* in Guangzhou since 2005.

**Methods:**

Based on the mosquito surveillance data using Mosq-ovitrap collection and human landing collection (HLC) launched at 12 sites in Guangzhou from 2015 to 2017, we established a MOI-based model of the basic dengue reproduction number (*R*_0_) using the classical Ross-Macdonald framework combined with a linear mixed-effects model.

**Results:**

During the survey period, the mean MOI and adult mosquito density index (ADI) using HLC for *Ae. albopictus* were 12.96 ± 17.78 and 16.79 ± 55.92, respectively. The *R*_0_ estimated from the daily ADI (ADI_D_) showed a significant seasonal variation. A 10-unit increase in MOI was associated with 1.08-fold (95% CI 1.05, 1.11) ADI_D_ and an increase of 0.14 (95% CI 0.05, 0.23) in the logarithmic transformation of *R*_0_. MOI-based *R*_0_ of dengue varied by month and average monthly temperature. During the active period of *Ae. albopictus* from April to November in Guangzhou region, a high risk of dengue outbreak was predicted by the MOI-based *R*_0_ model, especially from August to October, with the predicted *R*_0_ > 1. Meanwhile, from December to March, the estimates of MOI-based *R*_0_ were < 1.

**Conclusions:**

The present study enriched our knowledge about mosquito-based surveillance indicators and indicated that the MOI of *Ae. albopictus* could be valuable for application in estimating the *R*_0_ of dengue using a statistical model. The MOI-based *R*_0_ model prediction of the risk of dengue transmission varied by month and temperature in Guangzhou. Our findings lay a foundation for further development of a complex efficient dengue risk prediction system.

**Graphical Abstract:**

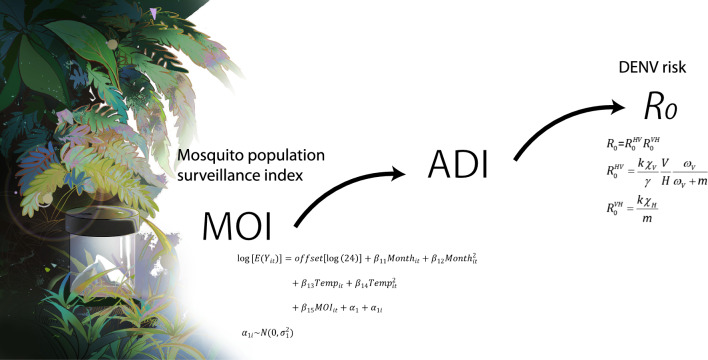

**Supplementary Information:**

The online version contains supplementary material available at 10.1186/s13071-024-06121-y.

## Background

Dengue fever, caused by dengue virus (DENV), is the most prevalent mosquito-borne disease in tropical and subtropical areas, with more than half of the global population living in areas with risk of dengue transmission [1, 2]. Due to human population growth, increase of population mobility, unplanned urbanization, expansion of dengue vectors, especially *Aedes aegypti* and *Ae. albopictus*, global warming, etc., the global burden of dengue has been increasing significantly in recent decades [3–7]. Since the re-emergence of a dengue outbreak in Foshan, Guangdong Province, in 1978, DENV has been epidemic in Southern China, especially in the provinces of Guangdong, Yunnan, Fujian and Zhejiang [8–11]. It is a real challenge regarding diagnosis, treatment, prevention and control of dengue infection because of the diversity of the clinical presentation [1]. The development of a novel vaccine and improvements in case management may improve the management, which still mainly depends on effective *Aedes* vector surveillance and control [12].

As *Aedes* biting data from human landing collections (HLCs) or human-baited double net traps (HDNs) in large-scale schemes are not available, *Aedes* surveillance systems based on ovitraps, immature stage surveys, adult mosquito trapping (BG Sentinel Trap, light trap or gravid female traps) and their correlated surveillance indicators [adult mosquito density index (ADI), Breteau index (BI), container index (CI), house index (HI), standard space index (SSI), eggs per ovitrap per week, female adults per sticky trap per week, etc.] have been widely performed in many epidemic areas [13–16]. In Guangzhou, the sentinel system has preliminarily been developed since 1978, though the indicators used for surveillance were very limited in the beginning. Since 2002, an *Ae. albopictus* surveillance net based on BI, HI and CI has been systematically established. Since 2005, an improved ovitrap named the mosquito and oviposition trap (Mosq-ovitrap) has been used to measure the mosquito or oviposition positive index (MOI) of the wild *Ae. albopictus* population in field surveillance [17].

Based on these tools and mosquito-based surveillance indicators, the establishment of an efficient dengue risk prediction system relying on indicator thresholds is helpful to effectively control the dengue epidemic. Using a logistic regression model combining data of the reported cases with the *Aedes* surveillance indices, BI = 5.1 and CI = 5.4 were suggested to control the epidemic efficiently with the fewest resources, and BI = 4.0 and CI = 5.1 were suggested to achieve effectiveness [18]. By comparing the relationship of the MOI and BI, Duan et al. developed an epidemic forecast and phased response system for dengue fever control and prevention in which MOI could be classified into four levels: < 5, 5–20, 20–40 and > 40 [19]. However, assessment is complex and not straightforward, so determining the relationship between mosquito abundance and dengue epidemics still faces challenges. On the one hand, the currently available information of *Aedes* population density indices may not predict risk for human infection (vector competence, etc.), which is influenced by environmental factors. On the other hand, larval/pupal indices (RI and BI) may not correlate directly with adult mosquito abundance.

In our previous studies, we systematically investigated and revealed the quantitative relationships between the ecological environmental factors and the biological behaviour parameters of *Ae. albopictus* populations including photoperiodic diapause incidence (DI), host-seeking density and route index (RI), which demonstrate the power of large-scale surveys combined with mathematical modelling [20–22]. Following this conception, the relationship between monitoring indicators (MOI as an example) and *Aedes* mosquito biting density can be effectively quantified through field surveys on a controllable scale, which can help connect the available *Aedes* population data collected in surveillance networks with dengue epidemic risk. In this study, based on the MOI data of *Ae. albopictus* collected from a 2-year, 12-site field investigation in Guangzhou, we established the MOI-based *R*_0_ model of dengue risk prediction under the Ross-Macdonald framework, which is valuable for development of a more convenient and effective risk assessment system to block dengue transmission.

## Method

### Study area and sampling sites

Like our previous studies [20–22], this study is part of a programme for monitoring and investigating the wild population of *Ae. albopictus*; the field experiments were conducted from 2015–2017 in Guangzhou, China (Additional file 1: Figure S1). Sanyuanli (SYL) in Yuexiu District, Jiahe (JH) in Baiyun District and Jiangpu (JP) in Conghua District in Guangzhou were chosen as the study areas, as in our previous work, representing three urbanisation levels: urban, suburban and rural settings (Additional file 1: Figure S1). Each setting included four land use categories: park, residential area, construction site and school (Additional file 1: Figure S1). Socioeconomic characteristics including population density and land structure of these three areas were described in our previous work [21].

### Mosquito surveillance by Mosq-ovitraps and Human landing collection

Mosquito surveillance by Mosq-ovitraps for each site was carried out once a month, based on the National Standard of Technologies of China [23]. A network of 600 Mosq-ovitraps was set up for 12 sites, with 50 at each study site. The traps were placed in the same locations each month. After 4 days in the field, the traps were brought to the laboratory and assessed for the presence of mosquito eggs, larvae and adults [17]. The *Aedes* MOI population index was calculated according to the following formulae: MOI = (number of ovitraps containing at least 1 *Ae. albopictus* adult or egg)/(ovitraps collected from the observation area) × 100 [17].

HLC for each site was carried out by two qualified operators for each site twice a month, based on the National Standard of Technologies of China [23]. Each operator gave their consent to carry out HLC after being informed of potential risks. Operators were attired in dark-coloured clothing and stood in the shade without direct sunlight. The collections were performed for 15 min. During the monitoring period, operators vigilantly monitored the exposed side of the leg. Biting female mosquitoes were caught with a mechanical aspirator as soon as they landed on the skin. Considering the schedule feasibility of conducting simultaneous multisite investigations long term in the field, HLCs were performed from 9:00–15:00 on each observation day. ADI was calculated by the follow formula: ADI = [number of female *Ae. albopictus* collected/(number of operators × (15 min/60 min) × number of observations].

### Estimate of *R*_0_ based on HLC

The Ross-Macdonald model, established in 1970, is considered the critical framework in risk assessment of mosquito-borne pathogen transmission [24]. It involves the whole progress of dengue transmission including susceptible, exposed, infectious and recovered conditions of humans and susceptible, exposed and infectious conditions of mosquitoes, as well as the mosquitoes' biting behaviour regarding humans [24]. In the Ross-Macdonald model framework, mosquito biting rates can be used to estimate the basic reproduction number (*R*_0_), which is defined as the total number of secondary infections produced by introducing a single infective case into a susceptible population (if *R*_0_ > 1, there is a risk for disease establishment in a certain area, while if *R*_0_ < 1, an introduced case may lead to a few new cases by chance, but the disease is not expected to establish or cause a large outbreak). We estimated *R*_0_ of DENV using a Ross-Macdonald model, and the formula for the estimation is as follows [24]:

$$R_{0} = R_{0}^{HV} R_{0}^{VH} ,\;R_{0}^{HV} = \frac{{k\chi_{V} }}{\gamma }\frac{V}{H}\frac{{\omega_{V} }}{{\omega_{V} + m}},\;R_{0}^{VH} = \frac{{k\chi_{H} }}{m}$$

where *k* is the human biting rate. *m* means mosquito mortality rate. ${\chi }_{V}$ represents the transmission efficiency from an infected human to mosquito, while ${\chi }_{H}$ indicates the transmission efficiency from an infected mosquito to human. ${1/\omega }_{V}$ is the length of the extrinsic incubation period. $1/\gamma$ indicates the infectious period in human hosts. $kV/H$ is the ratio of mosquitoes per human. To estimate $kV/H$, we first predicted the raw daily ADI (ADI_R_) with the hourly mosquito density, which was observed between 09:00 and 15:00 (described in Yin et al. [20]), based on a quasi-Poisson mixed-effects model (Additional file 2: Text S1). Then, we estimated the daily ADI (ADI_D_) by multiplying the predicted ADI_R_. The values of parameters and corresponding references are given in Additional file 3: Table S1.

To examine the temporal variations of ADI_D_ and *R*_0_, we used kernel regression to smooth the time series. A bootstrap method was used to estimate the 95% confidence intervals (95% CIs) of the smoothed ADI_D_ and *R*_0_.

### Associations of MOI with ADI_D_ and ***R***_***0***_

A quasi-Poisson mixed-effects model was used to assess the association between MOI and ADI_D_ as follows:

$$\begin{aligned} \log \left[ {E({\text{ADI}}_{{{\text{Dit}}}} } \right)] & = {\text{offset}}\left[ {\log \left( {24} \right)} \right] + \beta_{11} {\text{Month}}_{{{\text{it}}}} + \beta_{12} {\text{Month}}_{{{\text{it}}}}^{2} \\ & \quad + \beta_{13} {\text{Temp}}_{{{\text{it}}}} + \beta_{14} {\text{Temp}}_{{{\text{it}}}}^{2} \\ & \quad + \beta_{15} {\text{MOI}}_{{{\text{it}}}} + \alpha_{1} + \alpha_{1i} \\ & \alpha_{1i} \sim N\left( {0,\sigma_{1}^{2} } \right) \\ \end{aligned}$$

where ${{\text{ADI}}}_{{\text{Dit}}}$ is the daily ADI at the sampling site *i* at the time point *t*. Quadratic functions were applied to calendar month and monthly mean temperature. A linear function was used for MOI. ${\beta }_{11}-{\beta }_{15}$ and ${\alpha }_{1}$ are regression coefficients for calendar month, monthly mean temperature, MOI and intercept. ${\alpha }_{1i}$ is a random-effect intercept for investigation site.

A linear mixed-effects model was used to associate MOI and the logarithmic transformation of *R*_0_ as follows:

$$\begin{aligned} \log \left( {R_{{0{\text{it}}}} } \right) & = \beta_{21} {\text{Month}}_{{{\text{it}}}} + \beta_{22} {\text{Month}}_{{{\text{it}}}}^{2} \\ & \quad + \beta_{23} {\text{Temp}}_{{{\text{it}}}} + \beta_{24} {\text{Temp}}_{{{\text{it}}}}^{2} \\ & \quad + \beta_{25} {\text{MOI}}_{{{\text{it}}}} + \alpha_{2} + \alpha_{2i} \\ & \alpha_{2i} \sim N\left( {0,\sigma_{2}^{2} } \right) \\ \end{aligned}$$

where ${\text{log}}({R}_{0{\text{it}}})$ is the logarithmic transformation of *R*_0_ at the sampling site *i* at the time point *t*. The functions used for calendar month, monthly mean temperature and MOI were the same as in the model for assessing the association between MOI and ADI_D_.

We present the exposure-response curves of the associations between MOI and *R*_0_ by month, given the monthly mean temperature equals the average of monthly mean temperatures in Guangzhou during the study period.

### Sensitivity analysis

Sensitivity analysis was conducted to assess the association between MOI and *R*_0_ for two extreme scenarios in which minimum and maximum estimates of *R*_0_ were obtained by using the lowest or highest bound of parameters shown in Table 1. In addition, 0.1 and 0.2 (instead of 0.15) were added to ADI_9–15_ when associating ADI_R_ and ADI_9–15_.

**Table 1:** Parameters for estimating *R*_0_ in the Ross-Macdonald model

Parameter	Value (range)	References
*k*	0.09 (0.05, 0.16)	Manica et al. [25]
*m*	$$m= \left\{\begin{array}{ll}0.000114{T}^{2}-0.00427T+0.0639,& T\ge 15.0\;{^{\circ}{\text{C}}} \\ 0.5,& T<15.0\;{^{\circ}{\text{C}}} \end{array}\right.$$	Brady et al. [26]
${\chi }_{H}$	31% (10%, 50%)	Lambrechts et al. [27] Paupy et al. [28]
${\chi }_{V}$	31% (10%, 50%)	Lambrechts et al. [27] Paupy et al. [28]
${1/\omega }_{V}$	10 (7, 14) days	Nur Aida et al. [29]
$1/\gamma$	6 (3, 7) days	Manore et al. [30]
*X*	0.101	Manica et al. [25]
$kV/H$	Time-dependent	Estimated using a quasi-Poisson mixed model^a^

*T* temperature

^a^Described in detail in Additional file 3: Table S1

### Other data collection and analysis software

Temperature data were downloaded from the open database Guangzhou Climate Data Network (http://data.tqyb.com.cn/weather/index.jsp). All analyses and data visualisation were carried out using R (version 3.5.1.) software. The ‘mgcv’ package was used to fit the quasi-Poisson mixed-effects model and the linear mixed-effects model.

## Result

### Mosq-ovitrap and HLCs data

**Fig. 1:**
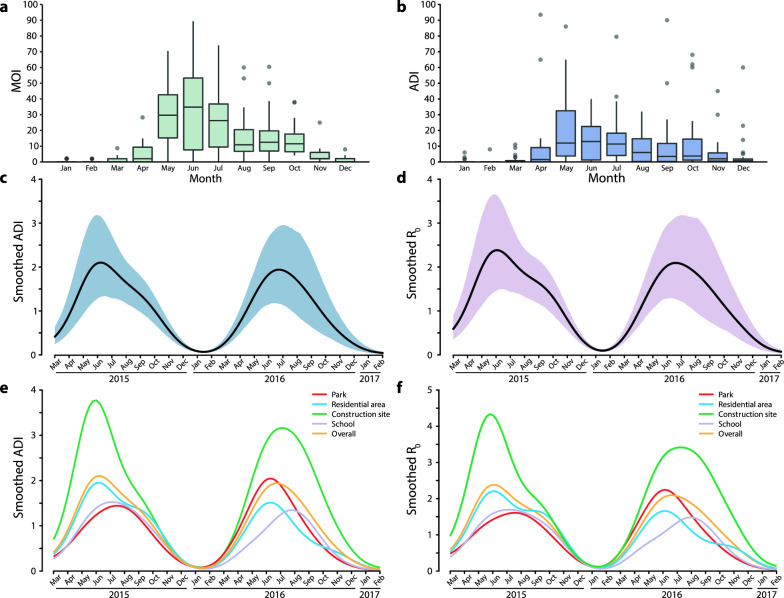
Estimates of ADI_D_ and *R*_*0*_ based on HLCs. **a**, **b** Seasonal variation of ADI (**a**) and MOI (**b**) in the wild population of *Aedes albopictus* in Guangzhou. Horizontal lines in the boxes show the median and the bars crossing the boxes show the maximum and minimum except outliers. Black dots show outliers, while 11 outliers are not shown in (**b**) for better visualization of the seasonal dynamic of ADI. **c**, **d** Temporal variations of the ADI_D_ (**c**) and *R*_0_ (**d**) between March 2015 and February 2017, Guangzhou. **e**, **f** Temporal variations of the ADI_D_ (**c**) and *R*_0_ (**d**) in four land use categories between March 2015 and February 2017, Guangzhou. In **c**–**f**, black lines represent the smoothed time series estimated with a kernel regression model. Orange and purple regions indicate the 95% confidence intervals of the smoothed time series of ADI_D_ and *R*_0_, respectively. ADI_D_ = daily ADI

During the survey period, 14,400 Mosq-ovitraps were utilized in the field investigation from March 2015 to February 2017 at 12 sites in Guangzhou (Fig. 1). We recovered 13,655 Mosq-ovitraps, and the total recovery rate was 84.83%. The eggs and adults caught by Mosq-ovitraps were mostly *Ae. albopictus* according to species identification. Of these, 1656 Mosq-ovitraps were found positive for *Ae. albopictus* adults or eggs. The calculated MOIs ranged from 0 to 89.36, and the mean MOI was 12.96 ± 17.78 (Fig. 1). The mean calculated *Ae. albopictus* ADI by HLCs in 15 min was 16.79 ± 55.92. Regarding the different land use categories (RES, PAR, CON and SCH) and urbanisation levels (urban, SYL; suburban, JH; rural, JP), both MOIs and ADIs in the wild population of *Ae. albopictus* in Guangzhou showed a consistent seasonal dynamic with an obvious active period from April to November (Fig. 1, Additional file 4: Figure S2).

Among the four land use categories, MOI and ADI had the following ranges and mean values: 0 to 70.5 and 0 to 161.5, respectively (mean values 11.9 ± 16.3 and 10.1 ± 23.7, respectively) in PAR; 0 to 65.2 and 0 to 144.0, respectively (mean values 9.6 ± 14.6 and 10.4 ± 21.3, respectively) in RES; 0 to 89.4 and 0 to 616.5, respectively (mean values 12.9 ± 19.4 and 41.0 ± 103.7, respectively) in CON; 0 to 75.0 and 0 to 30.0, respectively (mean values 15.5 ± 20.0 and 5.6 ± 8.1, respectively) in SCH (Additional file 4: Figure S2). At the three urbanization levels, these two indexes showed 0 to 70.5 and 0 to 34.0, respectively (mean values 9.5 ± 14.2 and 4.4 ± 6.7, respectively) in SYL; 0 to 83.0 and 0 to 161.5, respectively (mean values 15.3 ± 20.9 and 13.0 ± 24.6, respectively) in JH; 0 to 89.4 and 0 to 616.5, respectively (mean values 12.6 ± 17.2 and 33.0 ± 91.42, respectively) in JP (Additional file 4: Figure S2).

### Estimates of ADI_D_ and ***R***_0_ based on HLCs

Estimates of ADI_D_ of *Ae. albopictus* and ADI_D_-based *R*_0_ of dengue in 12 investigation sites in Guangzhou from March 2015 to February 2017 are presented in Fig. 1. The estimated MOI, ADI_D_ and *R*_0_ varied among investigation sites and months (Fig. 1, Additional file 5: Figure S3). Between March 2015 and February 2017, the overall smoothed ADI_D_ and *R*_0_ increased from January–February to June, peaking during June to July, and then decreased in general (Fig. 1). Higher ADI_D_ and *R*_0_ were observed in construction sites than in other ecological habitats (Fig. 1).

### Associations of MOI with ADI_D_ and ***R***_0_

MOI was positively associated with ADI_D_ and the logarithmic transformation of *R*_0_. Specifically, a 10-unit increase in MOI was associated with 1.08-fold (95% CI 1.05, 1.11) ADI_D_ (Table 2) and an increase of 0.14 (95% CI 0.05, 0.23) in the logarithmic transformation of *R*_0_ (Table 3).

**Table 2:** Results of the models assessing the associations between MOI and ADI_D_

Variable	*b*^a^	(95% CI)	exp(*b*)	(95% CI)	*P*
Month	1.16	(0.33, 1.99)	3.19	(1.40, 7.28)	0.006
Month^2^	− 0.08	(− 0.14, − 0.02)	0.92	(0.87, 0.98)	0.007
Temp	0.45	(0.13, 0.76)	1.57	(1.14, 2.14)	0.006
Temp^2^	− 0.08	(− 0.14, − 0.01)	0.93	(0.87, 0.99)	0.016
MOI	0.08	(0.04, 0.11)	1.08	(1.05, 1.11)	< 0.001
Intercept	− 1.04	(− 1.41, − 0.66)	0.35	(0.24, 0.52)	< 0.001

*ADI*_*D*_ daily ADI, *95% CI* 95% confidence interval, *Temp* monthly mean temperature. Month^2^ and Temp^2^ are quadratic terms of month and monthly mean temperature, respectively

^a^Regression coefficients of Temp^2^ and MOI were multiplied by 10, while the regression coefficient of intercept was divided by 10

**Table 3:** Results of the model assessing the association between MOI and the logarithmic transformation of *R*_0_

Variable	*b*^a^	(95% CI)	*P*
Month	1.71	(1.24, 2.18)	< 0.001
Month^2^	− 0.11	(− 0.14, − 0.07)	< 0.001
Temp	1.75	(1.43, 2.07)	< 0.001
Temp^2^	− 0.04	(− 0.04, − 0.03)	< 0.001
MOI	0.14	(0.05, 0.23)	0.003
Intercept	− 2.61	(− 2.93, − 2.28)	< 0.001

*b* Regression coefficient, *95% CI* 95% confidence interval, *Temp* monthly mean temperature. Month^2^ and Temp^2^ are quadratic terms of month and monthly mean temperature, respectively

^a^Regression coefficients of MOI were multiplied by 10, while the regression coefficient of intercept was divided by 10

**Fig. 2:**
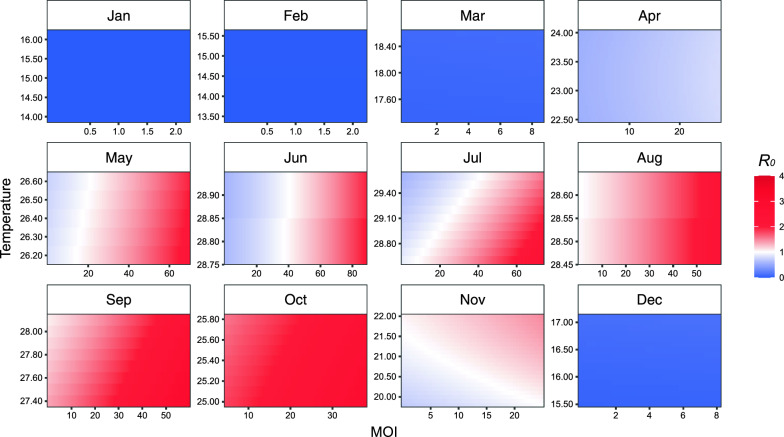
Color level plots of temperature, MOI and *R*_0_. The temperature ranges considered were the ranges of monthly mean temperatures observed in Guangzhou during the study period for each month, and the MOI ranges were what we observed at the 12 investigation sites

**Fig. 3:**
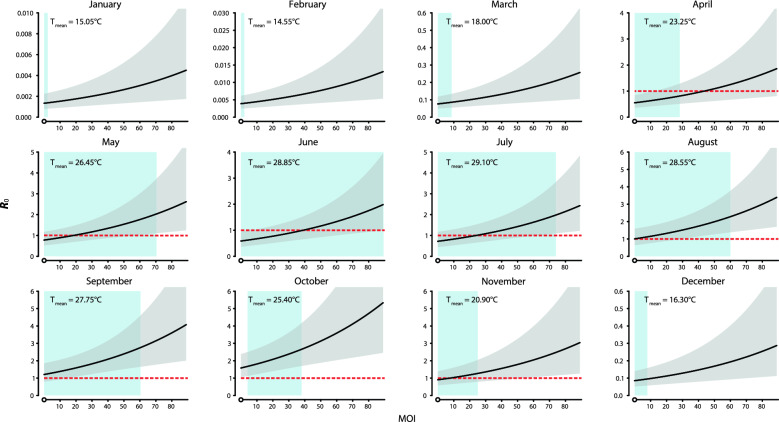
Exposure–response curves of the associations between MOI and *R*_0_. Lines represent the predicted *R*_0_, given temperature equals the average of monthly mean temperatures for each month. Grey regions represent the corresponding 95% confidence intervals of the predicted *R*_0_ estimated from the linear mixed-effects model. Blue regions indicate the observed ranges of MOI for each month

Figure 2 gives the color level plots of monthly mean temperature, MOI and estimated *R*_0_. Figure 3 presents the exposure-response curves of the associations between MOI and *R*_0_ by average of monthly mean temperature. The ranges of observed MOI largely increased during January to June and decreased afterwards, and the largest observed MOI was ≥ 60 between June and September. From April to July and in November, *R*_0_ was estimated to be > 1 when MOI was > 43.6, 18.5, 38.9, 24.0 and 7.6, respectively, given specific average monthly mean temperatures. The point estimates of *R*_0_ exceeded 1 during August and October. (Figs. 2 and 3).

### Model sensitivity analysis

Results of sensitivity analyses consistently indicated the positive associations between MOI and the logarithmic transformation of *R*_0_ (Additional file 6: Table S2).

## Discussion

A better understanding of mosquito-based surveillance indicators is critical to establish an efficient dengue risk prediction system. Our study indicated that MOI of *Ae. albopictus* could be a valuable mosquito surveillance indicator applied for estimating the *R*_0_ of dengue. Due to the different lifestyles of *Ae. albopictus* and *Ae. aegypti*, *Ae. albopictus* is characteristically peridomestic while *Ae. aegypti* is domestic. Multidimensional heterogeneity of *Ae. albopictus* habitats showed in larval/pupal surveys was complexly determined by inconsistent dynamics of total aquatic habitats ecosystem, *Ae. albopictus* adult oviposition behaviour and its diapause egg hatching [21]. The larval/pupal survey results were also limited by the investigators' experience. Thus, the application of CI, HI and BI in surveillance of *Ae. albopictus* has been unsatisfactory so far. However, the adult traps, such as BG-sentinel traps, are expensive to implement in large-scale schemes and all epidemic regions worldwide [31]. By contrast, MOI is a reflection of mosquito egg-laying behaviour, which is strongly correlated with the biting behaviour, which is also a more objective indicator of local mosquito density in the stable period in the investigation sites.

Based on the Ross-Macdonald theory, assessment of the complex and unclear relationship between the mosquito abundance index (MOI) and dengue epidemic was carried out using ADI as a bridge. Our previous study indicated that ADI varied across time points within a day [20, 21]. Therefore, we estimated the ADI for a whole day based on a model which related the ADI for a whole day and the ADI between 09:00 and 15:00 instead of using the ADI of a specific period to be the proxy of the ADI for a whole day. The climatic factor affects multiple parameters of the assessment. In our study, we tried to include the climatic factor directly in the variable parameter to eliminate the secondary effects on biting frequency by affecting population density and making the level of the model clearer to avoid blindly increasing the complexity of the model. Furthermore, instead of including sampling site in the model as a fixed-effect variable, we treated sampling site as a random-effect variable. The population-level results based on the models can indicate the overall situation in Guangzhou. However, such estimates may be too conservative (i.e. too high) in terms of dengue prevention and control, especially in areas with a high level of dengue transmission.

Concerning the predicted risk based on MOI, which varied by month, dengue transmission showed an obvious seasonal DENV epidemic pattern in Guangzhou. It showed *R*_0_ < 1 for nearly all MOIs and average monthly temperature conditions from November to April. Although the MOI values in April and November were similar to those in October, the *R*_0_ indicated low epidemic risk of DENV transmission for the lower average monthly temperature. The quantitative relation of low transmission risk in these months supported the beginning of DENV outbreaks in Guangzhou being mainly caused by imported cases [32, 33]. By contrast, in August, the point estimates of *R*_0_ exceeded 1 for an extremely wide range in this high average monthly temperature condition, which required powerful local mosquito control in Guangzhou. Furthermore, it is believed that multiannual cycles of the DENV epidemic took place across Southeast Asia and South China, which were highly coherent with the Oceanic Niño Index [34–36]. In years of El Niño, the increase in temperature will drive the increase in epidemic risk. Then, higher temperatures than multiyear average values in April and May will also cause the occurrence of an *R*_0_ value > 1 warning line to come early.

A standard index of transmission intensity and threshold criteria is critical for controlling mosquito-borne infections. As mentioned in the Introduction, some researchers have proposed using some index thresholds to prevent mosquito-borne disease transmission. In the Guangzhou surveillance net, the risk levels are determined by the stationary BI (< 5, 5–10, 10–20, > 20), MOI (< 5, 5–10, 10–20, > 20) and SSI (< 1.0, 1.0–1.5, 1.5–2.0, > 2.0) thresholds, respectively [19]. However, in our study, we found that the MOI thresholds associated with *R*_0_ = 1 varied across months as well as scenarios of different mean temperature. The current control and prevention trend requires precise disease risk estimation; the modeling proposal in this study can be applied to further establish a reliable automated intelligent surveillance and warning system of Aedes-borne diseases.

## Conclusions

In conclusion, our study indicates that the MOI of *Ae. albopictus* could be a valuable mosquito surveillance indicator applied for estimating the *R*_0_ of dengue with a statistical model. The MOI-based *R*_0_ model predicting the risk of dengue transmission varied by month in Guangzhou. Our findings could improve the development of an efficient risk prediction system for dengue outbreaks.

### Supplementary Information

**Additional file 1: Figure S1.** Study areas and surrounding environments of the selected investigation sites in Guangzhou. Sanyuanli (SYL, an urban area in Yuexiu District), Jiahe (JH, a suburban area in Baiyun District) and Jiangpu (JP, a rural area in Conghua District) represent the three urbanization levels. The green triangles, rohombus, square and pentagram indicate construction site (CON), park (PAK), residential area (RES) and school (SCH), respectively, corresponding to the four land use categories.

**Additional file 2: Text S1.** Estimated daily ADI using the observed mosquito density from 09:00 to 15:00.

**Additional file 3: Table S1.** Raw data of ADI and MOI from field investigation between March 2015 and February 2017, Guangzhou.

**Additional file 4: Figure S2.** Monthly dynamics of MOI and ADI from March 2015 to Feburary 2017 for four land use categories (CON, construction site; PAR, park; RES, residential area; SCH, school) in three urbanization levels (JH, Jiahe, a suburban area; JP, Jiangpu, a rural area; SYL, Sanyuangli, a urban area).

**Additional file 5: Figure S3.** Temporal variations of the MOI between March 2015 and February 2017, Guangzhou.

**Additional file 6: Table S2.** Results of models assessing the association between MOI and logarithmic transformation of *R*_0_ in the sensitivity analysis.

## Data Availability

The data from this manuscript are publicly available at https://gitee.com/xiangguo99/aedes-moi.

## Abbreviations

DENV: Dengue virus
MOI: Mosquito or oviposition positive index
*R*_0_: The basic reproduction number
HLC: Human landing collections
ADI: Adult mosquito density index
BI: Breteau index
CI: House index
HDN: Human-baited double-net trap
