# Supplementary material for: Modelling the dynamic basic reproduction number of dengue based on MOI of Aedes albopictus derived from a multi-site field investigation in Guangzhou, a subtropical region
Source: Parasit Vectors. 2024 Feb 21;17:79. doi: 10.1186/s13071-024-06121-y (PMC11325734; doi:10.1186/s13071-024-06121-y)
Supplement: Supplementary file 1 — Additional file 1: Figure S1. Study areas and surrounding environments of the selected investigation sites in Guangzhou. Sanyuanli (SYL, an urban area in Yuexiu District), Jiahe (JH, a suburban area in Baiyun District) and Jiangpu (JP, a rural area in Conghua District) represent the three urbanization levels. The green triangles, rohombus, square and pentagram indicate construction site (CON), park (PAK), residential area (RES) and school (SCH), respectively, corresponding to the four land use categories. [file 13071_2024_6121_MOESM1_ESM.pdf]

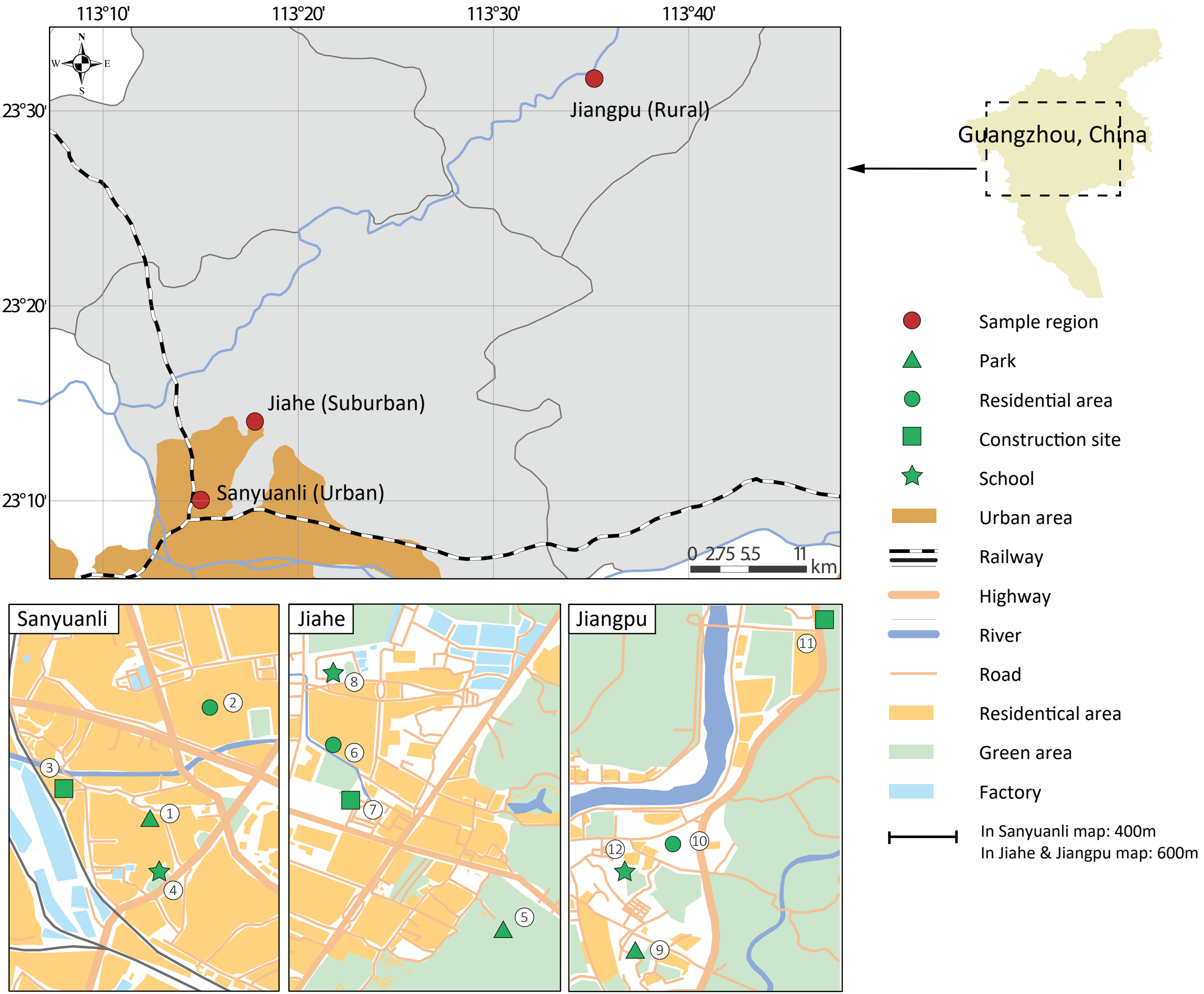
113°10'
113°20'
113°30'
113°40'
23°30'
23°20'
23°10'
N
S
W
E
Jiangpu (Rural)
Jiahe (Suburban)
Sanyuanli (Urban)
0 2.75 5.5 11 km
Guangzhou, China
Sample region
Park
Residential area
Construction site
School
Urban area
Railway
Highway
River
Road
Residentical area
Green area
Factory
In Sanyuanli map: 400m
In Jiahe & Jiangpu map: 600m
Sanyuanli
Jiahe
Jiangpu
1
2
3
4
5
6
7
8
9
10
11
12
