# Supplementary material for: Modelling the dynamic basic reproduction number of dengue based on MOI of Aedes albopictus derived from a multi-site field investigation in Guangzhou, a subtropical region
Source: Parasit Vectors. 2024 Feb 21;17:79. doi: 10.1186/s13071-024-06121-y (PMC11325734; doi:10.1186/s13071-024-06121-y)
Supplement: Supplementary file 2 — Additional file 2: Text S1. Estimated daily ADI using the observed mosquito density from 09:00 to 15:00. [file 13071_2024_6121_MOESM2_ESM.pdf]

Text S1

We used a quasi-Poisson mixed-effects model to examine the association between the raw daily mosquito density (ADI_R) and the hourly mosquito density which were observed during 09:00-15:00. The model is as follows:

$$\log[E(ADI_{Rit})] = offset[\log(24)] + \beta_1 Month_{it} + \beta_2 Month_{it}^2$$
$$+ \beta_3 \log(ADI_{9-15it} + 0.15)$$
$$+ \alpha + \alpha_i$$

$$\alpha_i \sim N(0, \sigma^2)$$

Where ADI_{Rit} is the raw daily mosquito density at the sampling site *i* at the time point *t*. A quadratic function was applied to calendar month and a linear function was used for the logarithm transformation of hourly ADI for 09:00-15:00 (ADI_{9-15}) plus 0.15 (the minimum non-zero ADI_{9-15} is 0.286). $\beta_1 - \beta_3$ and α are regression coefficients for calendar month, the logarithm transformation of ADI_{9-15} plus 0.15, and intercept. α_i is a random-effect intercept for investigation site.
