# Supplementary material for: Modelling the dynamic basic reproduction number of dengue based on MOI of Aedes albopictus derived from a multi-site field investigation in Guangzhou, a subtropical region
Source: Parasit Vectors. 2024 Feb 21;17:79. doi: 10.1186/s13071-024-06121-y (PMC11325734; doi:10.1186/s13071-024-06121-y)
Supplement: Supplementary file 3 — Additional file 3: Table S1. Raw data of ADI and MOI from field investigation between March 2015 and February 2017, Guangzhou. [file 13071_2024_6121_MOESM3_ESM.pdf]

Table S1. Results of models which were used to assess the association between the mosquito density during 09:00-15:00 and ADI_D.

Variable	$b^{\#}$	(95% CI)	exp(b)	(95% CI)	P
log($ADI_{9\text{-}15}$ + 0.15)	0.51	(0.34, 0.68)	1.67	(1.41, 1.97)	<0.001
Month	1.28	(0.62, 1.95)	3.61	(1.86, 7.02)	0.001
Month^2	-0.09	(-0.13, -0.04)	0.92	(0.88, 0.96)	<0.001
Intercept	-0.31	(-0.57, -0.05)	0.74	(0.57, 0.95)	0.026

Note. b = regression coefficient; 95% CI = 95% confidence interval; $ADI_{9\text{-}15}$ = hourly ADI during 09:00 to 15:00; ADI_D = daily ADI.

$^{\#}$ The regression coefficient of intercept has been divided by 10.
