# Supplementary figures and images for: Modelling the dynamic basic reproduction number of dengue based on MOI of Aedes albopictus derived from a multi-site field investigation in Guangzhou, a subtropical region
Source: Parasit Vectors. 2024 Feb 21;17:79. doi: 10.1186/s13071-024-06121-y (PMC11325734; doi:10.1186/s13071-024-06121-y)

**a**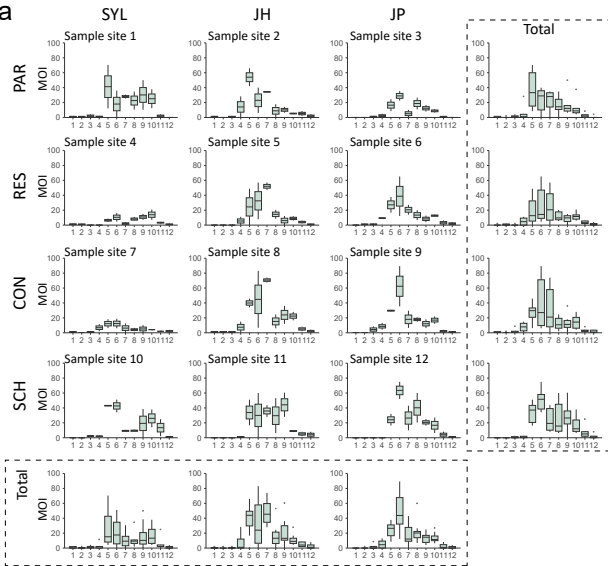**b**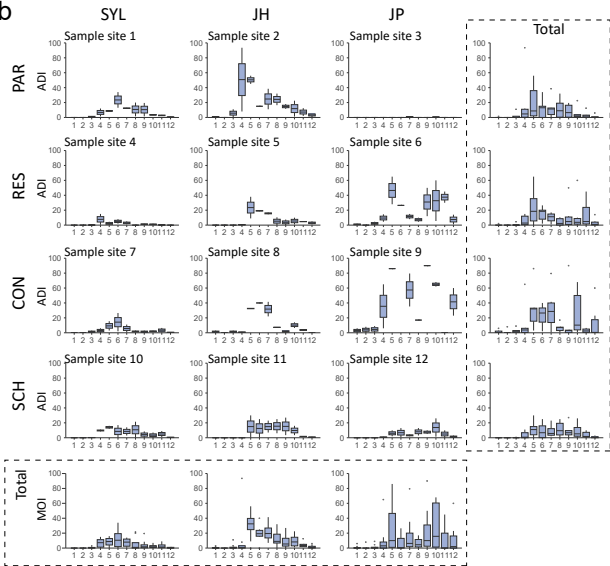

Supplement: Supplementary file 4 — Additional file 4: Figure S2. Monthly dynamics of MOI and ADI from March 2015 to Feburary 2017 for four land use categories (CON, construction site; PAR, park; RES, residential area; SCH, school) in three urbanization levels (JH, Jiahe, a suburban area; JP, Jiangpu, a rural area; SYL, Sanyuangli, a urban area). [file 13071_2024_6121_MOESM4_ESM.pdf]

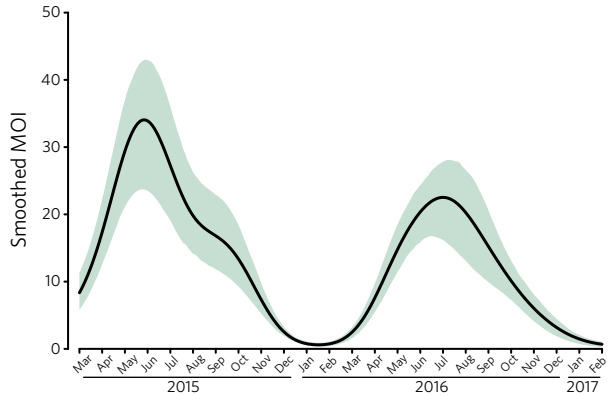

Supplement: Supplementary file 5 — Additional file 5: Figure S3. Temporal variations of the MOI between March 2015 and February 2017, Guangzhou. [file 13071_2024_6121_MOESM5_ESM.pdf]
