# Supplementary material for: Modelling the dynamic basic reproduction number of dengue based on MOI of Aedes albopictus derived from a multi-site field investigation in Guangzhou, a subtropical region
Source: Parasit Vectors. 2024 Feb 21;17:79. doi: 10.1186/s13071-024-06121-y (PMC11325734; doi:10.1186/s13071-024-06121-y)
Supplement: Supplementary file 6 — Additional file 6: Table S2. Results of models assessing the association between MOI and logarithmic transformation of R0 in the sensitivity analysis. [file 13071_2024_6121_MOESM6_ESM.pdf]

Table S2. Results of models assessing the association between MOI and logarithm transformation of R0 in the sensitivity analysis.

Sensitivity analysis	Variable	$b^{\#}$	(95% CI)	P
SA1	Month	1.73	(1.25, 2.21)	<0.001
	$Month^2$	-0.11	(-0.14, -0.07)	<0.001
	Temp	1.82	(1.49, 2.15)	<0.001
	$Temp^2$	-0.04	(-0.05, -0.03)	<0.001
	MOI	0.14	(0.05, 0.23)	0.003
	Intercept	-3.05	(-3.38, -2.72)	<0.001
SA2	Month	1.68	(1.22, 2.14)	<0.001
	$Month^2$	-0.11	(-0.14, -0.07)	<0.001
	Temp	1.68	(1.37, 1.99)	<0.001
	$Temp^2$	-0.04	(-0.04, -0.03)	<0.001
	MOI	0.14	(0.05, 0.22)	0.002
	Intercept	-2.35	(-2.66, -2.04)	<0.001
SA3	Month	1.68	(1.20, 2.16)	<0.001
	$Month^2$	-0.11	(-0.14, -0.07)	<0.001
	Temp	1.78	(1.46, 2.11)	<0.001
	$Temp^2$	-0.04	(-0.05, -0.03)	<0.001
	MOI	0.14	(0.05, 0.23)	0.003
	Intercept	-2.64	(-2.97, -2.32)	<0.001
SA4	Month	1.73	(1.26, 2.19)	<0.001
	$Month^2$	-0.11	(-0.14, -0.07)	<0.001
	Temp	1.73	(1.42, 2.05)	<0.001
	$Temp^2$	-0.04	(-0.04, -0.03)	<0.001
	MOI	0.13	(0.05, 0.22)	0.003
	Intercept	-2.58	(-2.90, -2.26)	<0.001

Note. b = regression coefficient; 95% CI = 95% confidence interval; Temp = monthly mean temperature. SA1 = minimum estimates of R_0 were obtained by using the lowest or highest bound of parameters shown in Table 1; SA2 = maximum estimates of R_0 were obtained by using the lowest or highest bound of parameters shown in Table 1; SA3 = 0.1 was added to $ADI_{9\text{-}15}$ when associating ADI_R and $ADI_{9\text{-}15}$; SA4 = 0.2 was added to $ADI_{9\text{-}15}$ when associating ADI_R and $ADI_{9\text{-}15}$.

The regression coefficients of MOI have been multiplied by 10, while the regression coefficients of intercept have been divided by 10.
